# How to quantify animal activity from radio‐frequency identification (RFID) recordings

**DOI:** 10.1002/ece3.4491

**Published:** 2018-10-02

**Authors:** Arne Iserbyt, Maaike Griffioen, Benny Borremans, Marcel Eens, Wendt Müller

**Affiliations:** ^1^ Behavioural Ecology and Ecophysiology Group University of Antwerp Wilrijk Belgium; ^2^ Department of Ecology and Evolutionary Biology University of California Los Angeles Los Angeles California; ^3^ Interuniversity Institute for Biostatistics and Statistical Bioinformatics (I‐BIOSTAT) Hasselt University Diepenbeek Belgium

**Keywords:** animal activity, blue tits, data processing, data validation, parental care, passive integrated transponder tags, radio‐frequency identification technology

## Abstract

Automated animal monitoring via radio‐frequency identification (RFID) technology allows efficient and extensive data sampling of individual activity levels and is therefore commonly used for ecological research. However, processing RFID data is still a largely unresolved problem, which potentially leads to inaccurate estimates for behavioral activity. One of the major challenges during data processing is to isolate independent behavioral actions from a set of superfluous, nonindependent detections. As a case study, individual blue tits (*Cyanistes caeruleus*) were simultaneously monitored during reproduction with both video recordings and RFID technology. We demonstrated how RFID data can be processed based on the time spent in‐ and outside a nest box. We then validated the number and timing of nest visits obtained from the processed RFID dataset by calibration against video recordings. The video observations revealed a limited overlap between the time spent in‐ and outside the nest box, with the least overlap at 23 s for both sexes. We then isolated exact arrival times from redundant RFID registrations by erasing all successive registrations within 23 s after the preceding registration. After aligning the processed RFID data with the corresponding video recordings, we observed a high accuracy in three behavioral estimates of parental care (individual nest visit rates, within‐pair alternation and synchronization of nest visits). We provide a clear guideline for future studies that aim to implement RFID technology in their research. We argue that our suggested RFID data processing procedure improves the precision of behavioral estimates, despite some inevitable drawbacks inherent to the technology. Our method is useful, not only for other cavity breeding birds, but for a wide range of (in)vertebrate species that are large enough to be fitted with a tag and that regularly pass near or through a fixed antenna.

## 1 INTRODUCTION

Automated monitoring of moving targets via radio‐frequency identification (RFID) technology is inextricably embedded in our human daily lives, with examples like theft prevention, stock management, pet identification, and access badges (Ngai, Moon, Riggins, & Yi, 2008). It is a low cost technology that allows instant reading and storage of large amounts of information without visual and physical contact. In its simplest form, a transponder (also called tag or microprocessor chip) can be attached to an object and signals its unique identification number via radio waves to a receiver antenna. RFID technology also created new opportunities for scientific research and quickly made its entrance into behavioral and ecological research from the early 1990s onwards. The monitoring of individual behavior via RFID applications has been applied successfully in a range of research areas including movement and foraging ecology (Bonter & Bridge, 2011), wildlife conservation (Dexter, Appleby, Edgar, Scott, & Jones, 2016), and social network interactions (Firth & Sheldon, 2015; Krause et al., 2013). A variety of study systems has been covered in doing so, including vertebrates (e.g., fish, salamanders, rodents, bats; Borremans et al., 2017; Charney, Letcher, Haro, & Warren, 2009; Kerth & Reckardt, 2003; Kobler, Humblet, Knaepkens, Engelen, & Eens, 2012) and invertebrates (e.g., bumblebees, beetles, ants; Molet, Chittka, Stelzer, Streit, & Raine, 2008; Robinson, Richardson, Sendova‐Franks, Feinerman, & Franks, 2009; Vinatier et al., 2010).

Birds appear to be a particularly suitable study system for the application of RFID technology (reviewed by Bonter & Bridge, 2011). Tags can easily be implanted under the skin or embedded in leg bands without long‐term effects on reproductive success or survival (Bonter & Bridge, 2011; Nicolaus, Bouwman, & Dingemanse, 2008; Schlicht & Kempenaers, 2015). Moreover, antennas can easily be integrated in artificial feeding stations, roosts, and breeding sites. RFID technology has largely reduced the constraints of traditional field observations and video monitoring, because RFID technology enables gathering substantial quantities of data with limited logistic restrictions related to battery life, memory for data storage, and is far less time‐consuming to obtain (Krause et al., 2013; Nomano, Browning, Nakagawa, Griffith, & Russell, 2014). Furthermore, large‐scale studies are possible for relatively small animals or animals in difficult terrain without interference from human presence. These numerous advantages have made RFID technology a very attractive tool for scientific research, but depending on the research question, a number of problems may arise.

In an attempt to improve the accuracy of the obtained behavioral estimates, our overarching aim is to provide an overview of the most common and important problems inherent to the RFID technology and to make well‐informed suggestions on how the negative impact of such problems can be limited. To this end, we use PIT (passive integrated transponder) tagged cavity breeding blue tits (*Cyanistes caeruleus*) providing food to their offspring as an empirical example. The automatic registration of feeding visits is fundamental to calculate behavioral estimates like individual investment and parental coordination in such cavity breeding birds (Johnstone et al., 2014), which is probably one of the most studied topics in behavioral and ecological research (Royle, Smiseth, & Kölliker, 2012). Nevertheless, the nature of the problems inherent to the RFID technology, as well as the possible solutions, likely extends to a wide range of study systems and experimental setups.

Missed registrations, and hence, underrated behavioral activity, are common and relate to at least six difficulties inherent to the technology. The chance of missed registrations by a tag decreases with (1) its signal strength and increases with both (2) the distance to the antenna and (3) the deviation from the ideal perpendicular angle between the tag and the registration zone. The chance of missed detections further increases with (4) the speed with which the animal, and hence, the tag, passes through the registration zone. Although the reader sample interval can usually be programed in accordance with the anticipated speed, it may be that animals move faster through the registration zone than the quickest possible scanning interval of the logger. Moreover (5), when two or more tags are simultaneously present in the registration zone, some logger types can register only one of them (Maselyne, Saeys, & Van Nuffel, 2015). Damage to the casing of the tag (6) is another rare problem that may result in absent signaling. Furthermore (7), although the simplicity of the data structure (i.e., tag ID, date and time for each passage) is often considered as an advantage, a lack of behavioral knowledge can result in incorrectly annotated activity. For example, a study may aim to understand feeding rates of a passerine bird visiting a feeder or a nest box. Problems would arise when a tagged bird is in the vicinity of an antenna, without taking food at an RFID‐equipped feeder or without bringing prey to the offspring in a nest box. Each registration would then result in erroneous overinterpreted behavioral activity. To the best of our knowledge, the implications of these potential problems have never been evaluated.

By far, the most important and largely unresolved/unstandardized problem is the way in which the data are processed. Most visits in the vicinity of an antenna result in more than one reading because a visit often takes longer than the programed reader sample interval (issue 4 above). As a consequence, data have to be trimmed to avoid superfluous registrations and thus highly overrated activity patterns. Thus far, three methods of data trimming have been applied in cavity breeding birds. The first method roughly quantified behavioral activity as the number of minutes with at least one registration by the antenna, relative to the total number of minutes of the registration period (David, Pinxten, Martens, & Eens, 2015; Patrick & Browning, 2011; Wilkin, King, & Sheldon, 2009). This method may result in overrated feeding visits when individual arrivals and exits occur in different minute slots or when birds hang on the rim of the entrance hole but do not enter. This estimate may also result in underrated provisioning behavior when birds deliver prey more than once within a given minute slot.

A more refined method is to apply an arbitrary cutoff time to erase all registrations that follow the preceding registration within a specified time period. This arbitrary decision is based on the assumptions that the cutoff time is shorter than both the expected time spent outside (c.f., refractory period; Johnstone et al., 2014) and inside the nest and may have large implications for parameter accuracy (see Discussion). For example, earlier studies with passerine birds applied a cutoff of 6 s (blue tits: García‐Navas, Ortego, & Sanz, 2009; Johnsen, Delhey, Schlicht, Peters, & Kempenaers, 2005) or 17 s (great tits, *Parus major*: Welbers et al., 2017). The remaining records were then divided by two to reach a visit rate for the given period. Although this procedure results in a better estimate for visit rates, a third method can further improve this estimate by determining the exact arrival time and by applying a longer, but well‐chosen cutoff time between consecutive recordings. In a study with cooperative breeding chestnut‐crowned babblers (*Pomatostomus ruficeps*), a 1‐min cutoff rule appeared optimal to isolate arrival times (Nomano et al., 2014). Any record of the same individual within 1 min was considered to be part of the same unique nest visit, while subsequent registrations with intervals longer than 1 min were regarded as independent visits. Although this method appeared suitable for males and helpers, this was not the case for females because their time spent in the nest was much more variable and generally longer (Nomano et al., 2014). Applying a shorter than optimal cutoff rule, however, would lead to an exponential increase of overrated registrations, because individuals that stay longer at the nest than the used cutoff time are counted twice. In contrast, applying a longer than optimal cutoff time would lead to an exponential increase of missed “true” visits, because fast individuals with foraging trips that are shorter than the cutoff time are discarded. This example illustrates that accurate identification of independent nest visits using RFID data will depend on optimizing the trade‐off between accepting nonindependent registrations and rejecting independent ones.

This third method determines exact arrival times, which further facilitates the quantification of additional behavioral parameters such as the coordination within social networks or coordinated feeding activities between pair members caring for their offspring. Arrival times allow to quantify estimates for behavioral coordination among cooperating individuals. Such behavioral estimates are important to reach a comprehensive understanding of the mechanisms promoting the diversity in cooperative behavior (Taborsky, Frommen, & Riehl, 2016), as supported by a growing number of empirical studies including birds (Johnstone et al., 2014; Mariette & Griffith, 2015), fish (Nowicki et al., 2018), and humans (Arueti et al., 2013).

Validation of the RFID technology with visual observations is crucial to ensure the accuracy of behavioral estimates, but such validations are limited to only a handful of studies with cavity breeding birds (Browning, Patrick, Rollins, Griffith, & Russell, 2012; García‐Navas et al., 2009; Lendvai et al., 2015; Nomano et al., 2014). Visit rates calculated from visual observations and simultaneous RFID monitoring gave relatively similar outcomes (range correlation coefficient (*r*) = 0.67–0.99 in the referred studies). Such studies, however, never reported the exact number of erroneous under‐ and overestimated registrations, while individual visit rate obtained via RFID technology may well be the balancing outcome of both errors. It remains yet to be validated whether behavioral parameters based on the exact timing of nest visits (behavioural coordinaton; e.g., alternation, Johnstone et al., 2014; e.g., synchronization, Mariette & Griffith, 2015), rather than the absolute number of visits can be correctly estimated from RFID technology (see also Nomano et al., 2014).

With our study, we illustrate how the above outlined difficulties can be tackled, using PIT tagged blue tits feeding their nestlings as a test case. We show how data can be processed based on the time spent on either side of the antenna. We validated the number and timing of nest visits obtained from the RFID technology by calibration against traditional video analyses, paying particular attention to over‐ and underrated visits. Ultimately, we aimed for a high comparability of behavioral estimates between both methods. Finally, we provide a guide of reference for future studies that wish to implement RFID technology in their study system, with the main goal of minimizing the effects of the abovementioned inherent problems. To this end, we provide a flexible and transparent code in both R (R core Team, 2015) and Excel visual basic (Microsoft^®^ Office) that is ready to use for many other species feeding their offspring. Our approach of data validation and data processing can be useful for a wide range of (in)vertebrate species that are large enough to carry a tag and that regularly pass near or through a fixed antenna (Whitham & Miller, 2016).

## 2 METHODS

### 2.1 Study system and RFID hardware

Fieldwork was performed for two consecutive years (2016 and 2017) between March and May in a nest box population of blue tits in Peerdsbos, a mature oak‐beech forest near Antwerp (51°16′N, 4°29′E, Belgium; Lucass, Iserbyt, Eens, & Müller, 2016). Blue tits use these artificial cavities to reproduce and to roost during winter. Nest boxes (*N* = 131) were checked twice per week for nest building, egg laying, and incubation. Nests were checked daily from the expected hatch date until hatching, here defined as day 0. Incubation is entirely restricted to females in blue tits (Nord & Nilsson, 2011). Sex roles remain after hatching, with female brooding behavior gradually decreasing as nestlings become endothermic (6–7 days; Andreasson, Nord, & Nilsson, 2016). The method described in this paper was approved and carried out in accordance with the guidelines of the Ethical Committee of the University of Antwerp, Belgium (ID: 2015‐64).

**Figure 1:**
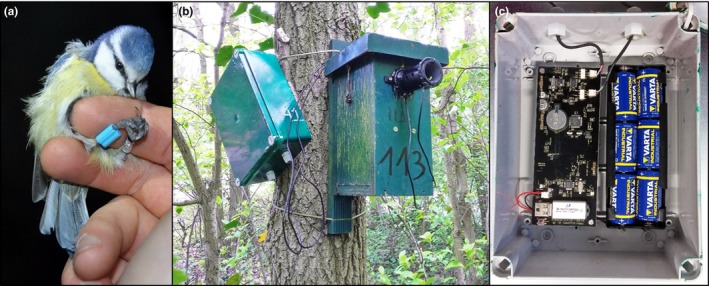
(a) An example of a PIT tag, here embedded in a leg band and attached to a blue tit. (b) Waterproof box containing the radio‐frequency identification (RFID) logger mounted on a tree (left side) with antennas placed in front of the nest box opening. (c) Inner side of the RFID box, containing the battery holder, RFID data logger circuit, and a 1 GB memory stick for data storage and programing

We made use of 2.6 mm plastic leg bands with PIT tags (EM4102, 125 KHz, Eccel Technology Ltd, Aylesbury, UK, Figure 1a), which were fitted after a bird was caught in its nest box while roosting in winter (both sexes), during incubation (females) or during the early nestling stage (day 6, males). PIT tags do not rely on an integrated power source, instead, PIT tags make use of the electromagnetic field generated by an antenna to signal its unique ID number. Such signals generally do not reach further than 20 cm. Birds could be caught repeatedly in subsequent years, and well‐functioning of the tag was always checked with a hand scanner (LID575, Dorset ID, Aalten, The Netherlands). The signal was lost in 3.4% of all repeated captures, presumably due to damage to the casing of older tags (>2 years). The core of our RFID system was protected within a waterproof box (Thermoplastic IP65 Junction Box, 220 × 170 × 80 mm; RS Components Benelux, Brussel, Belgium) and consisted of an electronic reader circuit (EM4102 data logger, Eccel Technology Ltd, Aylesbury, U.K.; Figure 1c). The logger was externally powered by six commonly used C‐type batteries (1.5V, LR14, Varta Industrial, Germany). These batteries lasted for at least 3 weeks of continuous outdoor recording with average temperatures ranging between minimum 5.0°C and maximum 18.5°C. The logger further contained two connections to independent antennas, and a USB memory stick (1 GB) for data storage and to program the minimal sample reading interval (250 ms), the date and the clock to the nearest second. The logger with both antennas (inner diameter: 40 mm) was installed around the nest box opening (Figure 1b), at least 2 days before the video recordings (see below). Metal was avoided in the vicinity of the antennas, because it may reduce the PIT tag signal strength. Installing the antennas was done in less than 2 min to ensure minimal disturbance. Without exceptions, all birds (*N* = 76) accepted this modification at their nest box and resumed provisioning behavior after 17.9 ± 2.8 min (range: 2.2–98.9 min).

### 2.2 Data processing and validation

To validate the RFID data with visual observations, infrared cameras (420TVL) were placed underneath the lid of the nest boxes (*N* = 20 in 2016; *N* = 18 in 2017) and continuously recorded between 7am and 5 pm when nestlings were 8, 9, or 10 days old. The first 30 min of video recordings were discarded to avoid a potential influence of human disturbance on bird activity. All videos were analyzed using the Observer XT program (version 10.5.572, 2011, Noldus Information Technology, Wageningen, The Netherlands). Entrance and exit times were scored to the nearest second for each individual until the least visiting parent had at least 10 visits or the analyses were terminated after 2 hr when one of the parents still did not reach the minimum number of required visits. A pilot study revealed that analyzing more visits did not significantly result in an improvement of individual visit rate (MG, unpublished results; see also Lendvai et al., 2015; Pagani‐Núñez & Senar, 2013).

**Figure 2:**
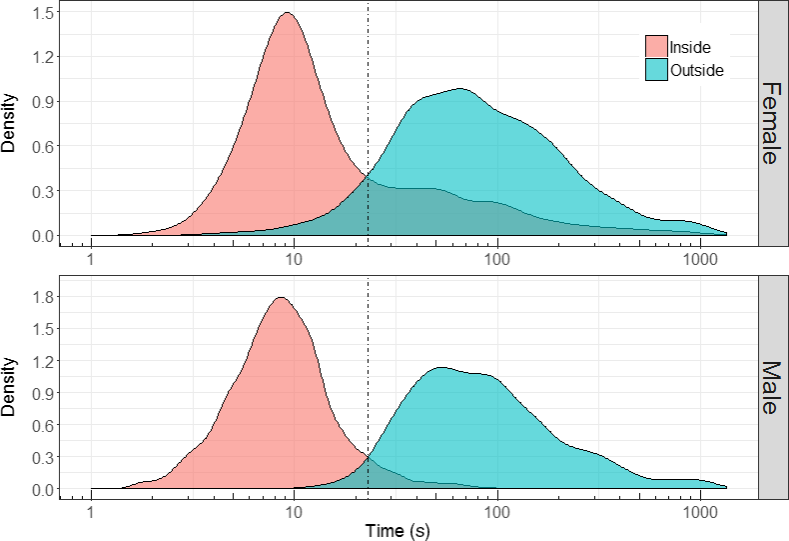
Density plots based on 1,852 video monitored female nest visits (top) and 1,823 male visits (bottom) from 38 nests when nestlings were 8–10 days old. The limited overlap between both density functions for time spent inside (red) and outside (blue) the nest box is visualized. The highest precision is reached when a cutoff of 23 s (dashed vertical line) is applied for both sexes to process RFID recordings. Note that the time on the *x*‐axis is log‐transformed for graphical clarity

The video analyses resulted in 1823 unique male records and 1852 female records, for which the time spent in‐ and outside the nest box was determined. Data density plots enabled us to quantify how often time periods in the nest box were longer than short foraging trips outside the nest box. Information about the overlap between these time periods is further important to isolate independent nest visits from a number of redundant, successive RFID registrations. The intersection of both density plots is interpreted as the most optimal, hence minimal, combination of erroneous double counts due long times spent in the nest box and erroneously deleted “true” visits due to very fast returns (Figure 2). We define this intersection as the optimal cutoff time, and it was determined for both sexes separately because differences in sex roles may occur (Lucass, Fresneau, Eens, & Müller, 2016).

The above procedure is, however, based on pooled data of all visual observations and does not account for any individual variation. We quantified the within‐ and between‐individual variation in time spent inside or outside the nest box with a univariate mixed modeling approach as detailed by Dingemanse and Dochtermann (2013). Sex was included as a fixed effect and birdID nested within nestID as a random effect to account for the nonindependence of male and female behavior within the same nest. Between‐individual variance explained only a small part of the total variance for both time spent in‐ and outside the nest box (11.2% and 6.7%, respectively). This low between‐individual variance allows to apply our cutoff value (determined by a subset of video‐monitored individuals) to large numbers of RFID‐monitored individuals.

The problems inherent to the RFID technology may frequently disrupt the pattern of incoming or outgoing signals (see above). In a subset of 242 parental visits (*N* = 10 nests), we noticed that only 86.8% and 43.8% of, respectively, all entries and departures were registered. The outer antenna registered 76.7% of all entries and 48.1% of all departures. Likewise, the inner antenna registered 48.1% of all entries and 58.5% of all departures. Therefore, we treated both antennas as a backup for each other by merging recordings from both. The original RFID data were sorted per ID number, and all successive readings with a time difference smaller than the cutoff were removed, independent of the registered antenna. Thus each unique visit had four chances to be registered by at least one antenna. This procedure enabled us to obtain the arrival time for each unique visit, although it is possible that only the departure time remains in case of speedy entrances. Based on these trimmed readings, we calculated behavioral activity for each ID as the number of nest arrivals per hour (i.e., nest visit rate). Nest visit rate is frequently interpreted as an estimate for parental investment during the nestling phase (Royle et al., 2012; for blue tits: Santema, Schlicht, Schlicht, & Kempenaers, 2017; Lucass, Fresneau, et al., 2016; Lucass, Iserbyt, et al., 2016). We then sorted the trimmed readings chronologically to calculate the level of coordinated provisioning within a pair (Johnstone et al., 2014). Parameters that rely on the exact timing of multiple individuals and may therefore be more susceptible for errors. Two concurrent estimates for within‐pair coordination are the level of alternated and synchronized number of nest visits (detailed in Bebbington & Hatchwell, 2016; Iserbyt, Fresneau, Kortenhoff, Eens, & Müller, 2017). Nest visit alternation was calculated as the number of male visits that followed female visits, and *vice versa*, relative to the total number of nest visits, minus one. Nest visit synchronization was calculated as the proportion of any (untrimmed) male and female registration that occurred within 10 s from each other, thereby assuming a visual encounter. These three behavioral parameters, nest visit rate, alternation, and synchronization, were calculated for each full sampling hour on each day of recording and could easily be pooled for longer time windows. Full scripts in R (R core Team, 2015) and Excel visual basic (Microsoft^®^ Office) are provided as Appendix S1.

Exact validation was done in two steps. First, the RFID data were trimmed as described above, and the resulting time series for male and female visits were aligned per nest with those of the simultaneously recorded video fragment. The exact number of overrated and missed registrations was counted, and both errors were expressed for each individual as percentages relative to the total number of “true” (video observations) visits. The three behavioral parameters, nest visit rate, alternation, and synchronization, were then calculated for the RFID dataset and the video dataset and compared with Pearson correlations in R (version 3.2.2; R core Team, 2015).

## 3 RESULTS

The least overlap between time spent in‐ and outside the nest box was 23 s and was equal for both sexes (see intersection of both density plots, Figure 2). We treated 23 s as the optimal cutoff time for reducing the superfluous number of successive RFID registrations. Applying this 23 s cutoff for data processing inherently generated an error of 2.4% (males) and 7.9% (females) of erroneously deleted “true” visits due to very fast returns (i.e., left of the 23 s cutoff in the density plots for time spent outside; Figure 2). The error due to overrating was considerably smaller for males that generally spent less time in the nest box (5.3%; right of the 23 sec cutoff; Figure 2) compared with females (25.8%), despite the similar peak for both sexes around 9 s in the density plots. Taken together, for 3.9% of all male visits and 16.8% of all female visits, one of both errors could be present, that is, either a double registration (long time in the nest box) or an erroneous deletion in case of a short nest return rate. Applying a smaller or larger cutoff than 23 s resulted in exponential changes in both opposite errors that canceled each other out to a large extent.

After RFID data processing, using the 23 s cutoff time, the male and female time series were matched with the time series of the corresponding video fragments. This resulted in 2.7% and 10.9% overrated registrations, and 13.2% and 18.2% missed registrations, respectively, for all male and female “true” visits (extremes excluded, see further). To a large extent, these errors correspond with those induced by the RFID data processing procedure.

**Figure 3:**
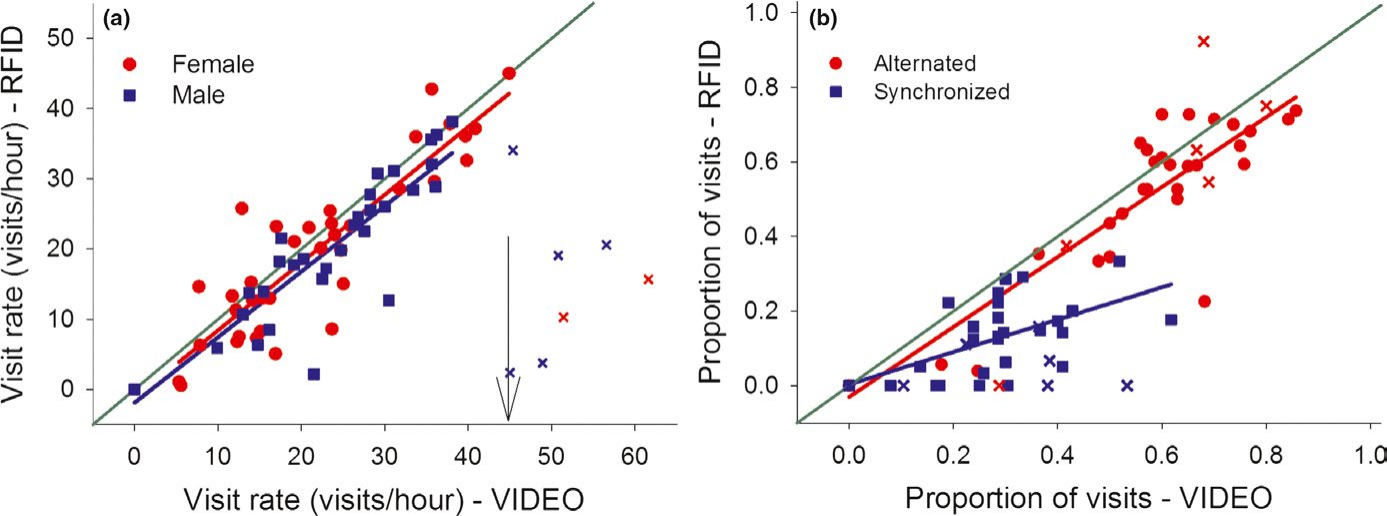
Radio‐frequency identification (RFID) data validation with the corresponding video data (*N* = 38) for three behavioral parameters. The thin green line in both panels exemplifies the most ideal situation where data by both methods are a perfect match. Panel a: male (blue squares) and female (red dots) visit rates, with the arrow pointing at 45 visits/hr, a threshold after which the RFID data becomes a poor match of the video data. Data generated by individuals with visit rates beyond this threshold are characterized by “*x*” in both panels. Panel b represents data validation for the proportion of alternated (red dots) and synchronized (blue squares) visits

Despite these overrated and missed detections, a highly significant correlation between visit rate based on video data and visit rate based on cleaned RFID data was found for both sexes (males: *r* = 0.41; females: *r* = 0.63; see Table 1, Figure 3a). Due to technical and/or biological reasons discussed below, visit rates obtained via RFID technology became a poor match of the “true” visit rates when the latter were higher than 45 visits per hour (see Figure 3a). This occurred in two females (5.2%) and five males (13.2%). After excluding these specific individuals, the correlation between both datasets became much stronger (males: *r* = 0.89; females: *r* = 0.90; Table 1). Similarly, RFID based alternation scores correlated well with the video based scores and remained very similar after excluding the individuals with high visit rates (Table 1, Figure 3b). Such validation was also significant for synchronization, although less strongly (Table 1, Figure 3b).

**Table 1:** Output of the Pearson correlations for each behavioral estimate calculated with the processed RFID data and the simultaneously recorded video data. The strength of these correlations is characterized by the correlation coefficient (*r*). Output presented between brackets is based on the full dataset (*n* = sample size), thus including individuals with more than 45 visits per hour

Parameter	*n*	*r*	*t*	*df*	*p*
Male visit rate	36 (38)	0.89 (0.41)	11.1 (4.91)	30 (36)	<0.001 (<0.001)
Female visit rate	32 (38)	0.90 (0.63)	11.4 (2.73)	34 (36)	<0.001 (0.0001)
Alternation	31 (38)	0.88 (0.88)	9.78 (11.1)	29 (36)	<0.001 (<0.001)
Synchronization	31 (38)	0.65 (0.53)	4.58 (3.77)	29 (36)	<0.001 (0.0001)

## 4 DISCUSSION

One of the major challenges inherent to the RFID technology is to isolate independent behavioral actions from a set of superfluous registrations (Nomano et al., 2014). Erasing successive registrations of a unique visit is based on an arbitrary threshold (cutoff) that only can be determined by visual observations in a subset of individuals. Visual observations for a subset of individuals are still needed, as it allows to quantify how much time is spent on average on the in‐ and outside of the nest box, and hence, on either side of the antenna. These visual observations should confirm a limited overlap between the data distribution of time spent within and outside the nest box (in our case study: males, 3.9%; females, 16.8%). We found the least overlap at 23 s for both sexes, which was further used as cutoff value to clean successive RFID readings. During RFID data processing, this optimal cutoff minimizes erroneous double counts due long times spent in the nest box and erroneously deleted “true” visits due to very fast returns. The cutoff value thus allows to isolate independent nest visits with maximized certainty.

Our data cleaning procedure is also applicable for other blue tit populations, given the similarity in both peaks of the data distributions between our and a German blue tit population (time spent in the nest box: 8–15 s; time spent between visits: 40–100 sec; Santema et al., 2017). The cutoff time may further be applicable for great tits as well, since average time spent in the nest box is less than 30 s in >90% of all great tit visits (Wilkin et al., 2009) and the time between visits peaks around 90 s (Johnstone et al., 2014; Schlicht, Santema, Schlicht, & Kempenaers, 2016). This suggests that our cutoff rule may be applicable for other studies that aim to quantify provisioning behavior of great and blue tits which are important model species in behavioral ecological research, yet validation is advised. A similar data cleaning procedure could easily be applied to other study systems, with specific examples including RFID‐equipped pet doors (Own, Shin, & Teng, 2013), bumblebee hives (Streit, Bock, Pirk, & Tautz, 2003), rodent passages (Schaefer & Claridge‐Chang, 2012), and even submerged lobster burrows (Aguzzi et al., 2011).

We urge future studies to determine not only the optimal (potentially sex‐specific) cutoff for data processing in their model species, but also to carefully consider the applied method for calculating behavioral estimates. For example, applying a shorter than optimal cutoff rule would lead to an exponential increase of overrated visits, because individuals that stay longer at the nest than the used cutoff are counted twice (Lendvai et al., 2015). That happens in 90.0% of all visits when a cutoff of 6 s (blue tits; García‐Navas et al., 2009) is applied to our video data, and 58.2% of all visits when a cutoff of 17 sec (great tits; Welbers et al., 2017) is used. These cited studies divided the processed registrations by two to calculate individual visit rates. However, this method would benefit from a lower cutoff (e.g., 2 s) so that each visit is indeed registered twice (99.8% when applied to our data). If this is not done, this method will result in overrated nest visits, a problem visualized by Lendvai et al. (2015) in their Figure 1. On the other hand, applying a longer than optimal cutoff rule would lead to an exponential increase of missed “true” visits, because fast individuals with intervisit intervals that are shorter than the applied cutoff are discarded during the data cleaning procedure. For example, applying a 120 s cutoff rule (blue tits; Schlicht & Kempenaers, 2015) to our data does result in a loss of 83.5% of all true visits.

After RFID data processing, we validated the behavioral parameters by calibration against the video observations and specifically identified errors due to over‐ and underrating. The latter has not explicitly been quantified in any previous study. We found 2.7% and 10.9% overrated registrations and 13.2% and 18.2% missed registrations for all validated male and female visits. Nevertheless, individual nest visit rates were strongly and positively correlated between the RFID and video data (males: *r *= 0.89; females: *r *= 0.90; see also Browning et al., 2012; García‐Navas et al., 2009; Lendvai et al., 2015; Nomano et al., 2014). However, for seven exceptionally fast individuals with more than 45 visits per hour that were excluded, the PIT registrations strongly lagged behind the true visits. The actual cause for this increase in missed detections remains elusive, but may relate to the speed of a tagged individual, combined with its angle through the antenna registration zone. We therefore recommend researchers to consider the passage speed and activity pattern of their model species before applying RFID technology. It is nevertheless unlikely that these exceptionally fast individuals would cause a problem in our data set. In undisturbed conditions, the average male and female visit rate with 8–10 day old nestlings in our population is 19.8 ± 0.57 visits per hour (mean ± *SE*;* N* = 36 control nests in 2017; AI unpublished results). An individual visit rate higher than 45 visits per hour never occurred for more than two consecutive hours. The exceptionally high visit rates observed here could be due to the disturbance during the installation of the video equipment. Parents often stay away from the nest following such a disturbance, causing elevated hunger of the nestlings that is compensated via particularly high parental visit rates upon return (Fresneau, Iserbyt, Lucass, & Müller, 2018).

The level of coordination among cooperating individuals is expected to correlate with the fitness payoffs for all individual team members (Arueti et al., 2013; Mariette & Griffith, 2015; Nowicki et al., 2018; Taborsky et al., 2016). However, estimates for coordinated behavior like alternation and synchronization (Bebbington & Hatchwell, 2016; Iserbyt et al., 2017) may be more sensitive to errors. Such parameters rely on the exact timing of visits and missed‐ and overrated registrations for each involved individual have accumulating effects. Nevertheless, we confirmed a high accuracy for alternation (*r *= 0.88) and high to moderate accuracy for synchronization (r = 0.65). The data fit for synchronization was flatter and under the ideal scenario (Figure 3b), which indicates an underestimation of this parameter via the RFID data, especially for pairs with high synchronization scores. The lower accuracy for this parameter may be explained by a relatively high probability of a missed incoming or outgoing registration for each visit. This may have underestimated the overall number of male and female overlaps within a time window of less than 10 s, despite having two antennas that served as a backup for each other. A comparison with previous studies is again hampered by the limited number of studies that validated their data. RFID data validation of parameters that rely on timing of nest visits is in fact limited to only one study in cooperative breeding babblers (Nomano et al., 2014). They report a high degree of accuracy for nest visit synchronization for all group members, except for the breeding female. Accurate estimations for females still required visual observations due to the high variability in time spent in the nest (Nomano et al., 2014). This again highlights the importance of sex‐specific RFID data validation as an essential first step for any study that applies this technology.

Besides incorrect RFID data processing, several other problems may further reduce the accuracy of behavioral estimates when using this technology. In cavity breeding birds, the signal strength of the tag in relation to the registration zone cannot be improved, because the individuals have to pass through the antenna loop to enter the nest box. However, strategic placement is recommended when point antennas are used, for example, when integrated in feeding stations (Farine, Aplin, Sheldon, & Hoppitt, 2015) or roost sites (Kerth & Reckardt, 2003). Underrated visits may be reduced by integrating the antennas into a structure, so that tagged individuals may be forced to pass the antenna under a more favorable perpendicular angle, and meanwhile reducing speed of the passage. Failure due to damaged plastic casings is less likely when subcutaneous injected glass PIT tags are used (Nicolaus et al., 2008), but these are less user friendly and may be more intrusive to the animals. We frequently observed different tag ID's within the same second. Thus, our RFID equipment was able to register PIT tags that were simultaneously present in the registration zone. We advise to contact the supplier about such limitations before purchasing the equipment (Maselyne et al., 2015). While the above issues may result in missed registrations, a lack of behavioral knowledge can result in an overestimated number of registrations. For example, it may occur that birds hang on the antenna on the in‐ or outside of the entrance hole, without passing through it. Similarly, birds can sit within the range of an RFID‐equipped feeder, without food uptake. Visual observations should therefore first determine the frequency of such overrated registrations.

For two reasons, we conclude that visual observations remain necessary in a subset of individuals for any study system. First, visual observations allow to determine the most optimal cutoff value, which is necessary to process RFID data. Second, the accuracy of behavioral estimates based on RFID technology should always be validated by calibration against visual observations. Once validated, the benefits of continuous monitoring via RFID for long time windows, with little disturbance and logistic constraints might vastly outweigh its limitations for a variety of scientific questions. Here, we used individually tagged blue tits during the nestling phase as a model system. Yet, given that our suggested procedure is based on two parameters inherent for many other study systems, that is, the time spent on either side of the antenna (Whitham & Miller, 2016), we suggest its applicability to a wide range of (in)vertebrate species.

## AUTHORS’ CONTRIBUTIONS

AI conceived the ideas and designed the methodology; AI and MG collected the data; AI, MG, and BB analyzed the data; AI led the writing of the manuscript. All authors contributed to revising the manuscript and gave final approval for publication.

## DATA ACCESSIBILITY

Data available from the Dryad Digital Repository: https://doi.org/10.5061/dryad.rr0v479
